# An automated illumination system for high-throughput photopharmacology studies: a case study of ROS-sensitive Zn- and Pd-phthalocyanine-loaded liposomes

**DOI:** 10.1039/d5an00927h

**Published:** 2025-10-20

**Authors:** Ali Eftekhari, Olga Lem, Alexander Efimov, Timo Laaksonen, Nikita Durandin

**Affiliations:** a Tampere University, Engineering and Natural Science, Materials Science and Environmental Engineering Tampere Finland nikita.durandin@tuni.fi ali.eftekhari@tuni.fi; b University of Helsinki, Faculty of Pharmacy, Division of Pharmaceutical Biosciences Helsinki Finland timo.laaksonen@helsinki.fi

## Abstract

High-throughput approaches for studying light-activated compounds are in high demand in biomedical applications. In this work, we designed and validated a cost-effective illumination platform that is easy to fabricate, customizable, and suitable for high-throughput *in vitro* studies. We demonstrated the performance of our system using a comparative study of reactive oxygen species (ROS)-sensitive liposomes loaded with two structurally identical phthalocyanines differing in their central metal, namely zinc and palladium. We showed that our system allows screening of a large set of chemical parameters in a short period of time for the optimization of light-triggered drug delivery systems, such as dye loading, power density, light dosage, and aerobic/anaerobic environment. Upon optimization, Pd(ii) phthalocyanine-loaded liposomes released up to 100% of calcein, while Zn(ii) phthalocyanine-loaded liposomes achieved only 50% release under the same conditions, *i.e.*, 690 nm incident light and 10 J cm^−2^ light dosage. Under anaerobic conditions, the calcein release was markedly reduced for both liposomes, confirming its ROS-dependent nature. The illumination system performed reliably throughout the study.

## Abstract 2

An automated illumination system for high-throughput photopharmacology studies has been developed and validated.

## Graphical abstract

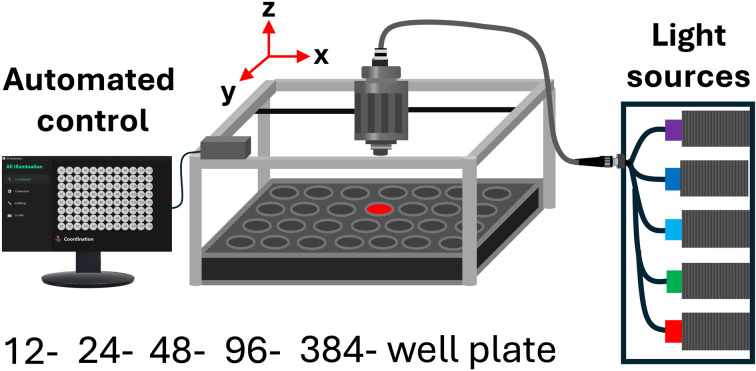

## Introduction

As an external triggering stimulus for drug release and drug activation, light serves as a versatile tool, offering precise control over both wavelength and intensity. By adjusting the beam's direction and modulating its intensity, illumination can be selectively delivered with precise spatial and temporal control, enabling the illuminated area to be defined and tailored to specific experimental and therapeutic needs.^1^ Approaches utilizing the physical properties of light have been explored to achieve controlled drug deposition and drug activation. Such approaches often rely on the distinctive light-responsive properties of photosensitive compounds or nanostructures.^2^

The discovery of new light-activated compounds for chemical biology and drug release applications demands high-throughput (HTP) solutions in order to identify lead compounds out of thousands of molecules in a research pipeline,^3,4^ a paradigm pioneered in the late 1980s. These techniques now underpin rapid advances across materials science—from energy storage to polymer libraries and 3D bioprinting—by running numerous experiments simultaneously to accelerate optimization and minimize reagent use.^5–7^ However, implementing HTP in photochemical and photobiological studies requires specialized illumination hardware—yet commercially available systems with both high-throughput capability and wavelength tunability typically carry substantial price tags and offer limited customization, creating barriers to progress in biomedical research.

To address these challenges, several research groups have developed custom-made illumination platforms. Bonnet *et al.* designed and fully characterized an LED-based cell irradiation device and the accompanying protocol for *in vitro* testing of photopharmacological compounds in standard 96-well plates, enabling controlled temperature and light intensities at 455, 520, and 630 nm under consistent dark conditions. They demonstrated its utility by assessing blue, green, and red-light cytotoxicity across six human cancer cell lines, thereby highlighting the importance of standardized, reproducible irradiation setups. Also, Spring *et al.* developed an open-source, LED-array-based photodynamic therapy (PDT) platform featuring a modular design for easy customization of LED type and wavelength, an integrated water-cooling loop for stable LED output, and robotic actuators to automate plate movement for hands-free operation. They also implemented pulse-width modulation (PWM) to achieve precise, linear control over array irradiance and provided both hardware and software details to enable researchers to build their own systems.^8,9^ To further improve customization for HTP assays, we designed a system that enables selective, well-by-well illumination with user-defined exposure time, a broad power density range, and light dosage. The setup accommodates multiple plate formats (12-, 24-, 96-, and 384-well) and uses an iris to match the beam diameter to the well geometry. The fiber-coupled optical path accepts any laser, LED, or diode laser that can be coupled into an optical fiber.

In addition to throughput and cost-effectiveness, careful selection of wavelength is another essential consideration when designing illumination setups for photopharmacological HTP assays. Wavelength selection is critical for both *in vivo* and *in vitro* applications. Ultraviolet (UV) and visible light irradiation can be harnessed for optogenetic control and to trigger drug release *via* various photochemical mechanisms, including photo-induced molecular transformations^10^ – such as bond cleavage,^11^ photoisomerization,^12^ and photocrosslinking.^13^ However, UV radiation has been classified as a biological mutagen.^14,15^ One way to overcome this issue is to employ visible light, which is generally regarded as non-toxic to cells.^16^ Utilization of visible light has led to important advances in cancer photochemotherapy, including photodynamic therapy (PDT) and photoactivated chemotherapy (PACT), approaches in which visible light irradiation is employed to selectively activate therapeutic agents within cancer cells.^17,18^

Within the visible range, red and far-red wavelengths are especially advantageous due to their deep tissue penetration.^19^ While wavelength remains a key factor in inducing cargo release, the irradiation modality—continuous wave (cw) *versus* pulsed light—also plays a critical role. Due to lower cost, simpler instrumentation, and reduced risk of phototoxicity, cw light irradiation (*e.g.*, LEDs or laser diodes) is generally favoured over high-peak-power pulsed light irradiation for most drug-release applications.^20^ Employing cw red/far-red light irradiation as a non-invasive and safe trigger for cargo release requires specially designed delivery systems and triggering molecules capable of absorbing this light.^21,22^ Porphyrinoids have frequently been employed for this purpose, due to their high absorption coefficients and excellent stability against bleaching.^23–26^ In the vast majority of studies, light triggering has been induced *via* photothermal or photooxidation mechanisms using specially designed nanocarriers.^26,27^ Among various drug delivery systems, liposomes serve as versatile vehicles capable of encapsulating both hydrophilic and hydrophobic drugs or triggering molecules. Porphyrinoids as well as other photosensitizers (PS) can be incorporated either within the lipid bilayer or encapsulated inside the liposomal core.^25^ Encapsulation of hydrophobic PS effectively addresses their solubility challenges, enhances monomerization and consequently improves their triggering activity. Additionally, liposomal drug delivery systems enable therapeutic effects by coupling photooxidation with the controlled release of the active pharmaceutical ingredient (API). To achieve such synergy, the liposomal bilayer must contain unsaturated lipids, which are sensitive to the reactive oxygen species (ROS) generated during illumination. Numerous studies have investigated the co-loading of photosensitizers (PS) with various small and large therapeutic agents in ROS-sensitive liposomes to enhance spatiotemporal control of drug release at the target site.^25,26^ ROS-sensitive, light triggered liposomal formulations for cancer therapy were thoroughly reviewed recently,^26^ and we gladly guide the curious reader in that direction. Although the overall processes of lipid oxidation and cargo release are fairly well characterized, the precise role of the central metal ion in porphyrinoid compounds in governing release efficiency is still not fully understood.

This study pursued two primary objectives. The first objective was to design and develop a fully customized, high-throughput, and cost-effective illumination system equipped with Python-based control software. This system allows precise, reproducible illumination of samples in standard microplate formats—including 12-, 24-, 48-, 96-, 384-well plates—with adjustable illumination diameter and controlled temperature. It also supports selective, well-by-well illumination across the plate, enabling targeted exposure of user-defined wells. Depending on the application, the system is adaptable to essentially any fiber-coupled light source—laser, LED, or diode laser—with selectable wavelength. Power density (irradiance) can be controlled in two ways: (i) manually, *via* the laser's current control knob, and (ii) automatically, *via* a Python-controlled high-precision, large-core fiber (400 μm) variable attenuator compatible with any fiber-coupled light source. Here, we demonstrated its performance with a 690 nm laser—delivering controlled light intensity within a consistent dark environment. The second objective was to utilize this device to investigate and compare photo-triggered drug release from liposomes loaded with two different photosensitizers, highlighting its practical utility for photochemical and photobiological research.

## Results and discussion

**Fig. 1:**
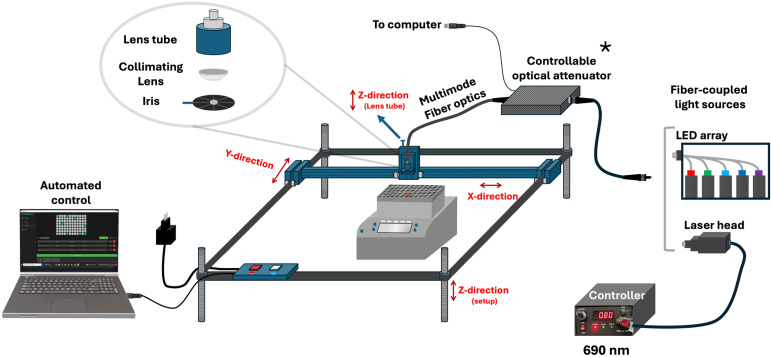
Custom, cost-effective high-throughput illumination setup. Light from a fiber-coupled laser—operating at either 690 nm or 808 nm—is passed through a variable iris diaphragm and collimating lens assembly. The system adjusts standard microplates on a temperature-controlled thermoshaker enabling reproducible, well-by-well light delivery and internal dark controls. The system was fully controlled *via* home-made Python-based Illumination software with 0.1 mm positioning accuracy in the *X*–*Y* direction. A photograph and a video of the fully enclosed, light-tight setup are provided in the SI. *Optical fiber attenuator enables automated irradiance control; not used in this study.

**Fig. 2:**
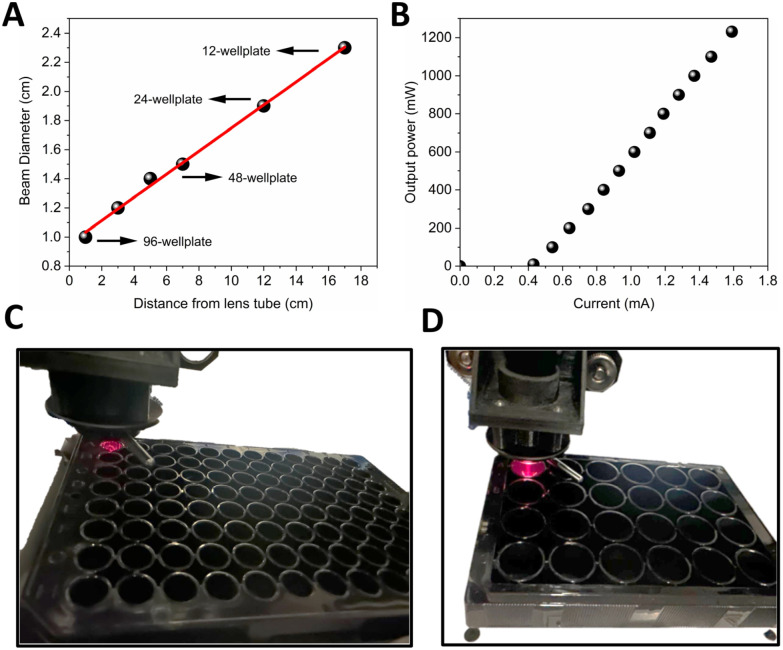
(A) Beam diameter over distance from the lens tube. (B) Output power *vs.* current. (C and D) 96 well plate and 24 well plate during illumination.

*In vitro* evaluation of light-triggered drug release demands highly reproducible irradiation under precisely controlled conditions, alongside a platform that is cost-effective, easily fabricated, customizable, and allows high-throughput studies. To meet these needs, we developed a custom illumination setup (Fig. 1) compatible with standard well plates. A single 96-well plate was mounted on a temperature-controlled thermoshaker, ensuring uniform thermal conditions for all samples. A fiber-coupled laser (selectable 690 nm or any light source capable of coupling into an optical fiber) delivers light through an adjustable iris and collimating optics. Precision *X*–*Y* stages—driven by our Python-based AIS Illumination software—navigate the laser head with 0.1 mm positional accuracy to irradiate only user-selected wells, while neighboring unselected wells remain dark as internal controls under identical conditions. Beam diameter and laser output power (at 690 nm) were measured, as shown in Fig. 2. As shown in Fig. 2A, at 1 cm below the lens tube, the beam diameter is ∼10 mm, which is well matched to a single well in a 96-well plate. By increasing the sample distance (*Z*), the beam expands to ∼ 24 mm, sufficient to cover larger wells in 24- and 12-well plates. For smaller formats (*e.g.*, 384-well plates), the integrated iris can be closed to ≤5 mm to reduce the spot size. Laser output power can be adjusted manually *via* the current control knob, spanning from 10 mW to 1200 mW at the fiber output (Fig. 2B). Alternatively, power can be automatically regulated by the control software through the inline large-core fiber variable attenuator, enabling precise irradiance delivery at the sample.

As photosensitizers, we chose two similar phthalocyanines. Palladium phthalocyanines are photoactive and often used as photosensitizers to achieve high generation of reactive oxygen species.^28,29^ They have demonstrated notable anti-inflammatory, antimicrobial, antitumor, antiviral, and antifungal activities, as well as good effectiveness in promoting drug release from liposomal carriers.^28,30,31^ However, palladium is a rare and expensive metal.^32^ Consequently, palladium phthalocyanines (PdPcs) are rarely explored in clinical studies and are used primarily in materials science, antimicrobial PDT, and early-stage cancer PDT research.^26,30,33,34^

**Fig. 3:**
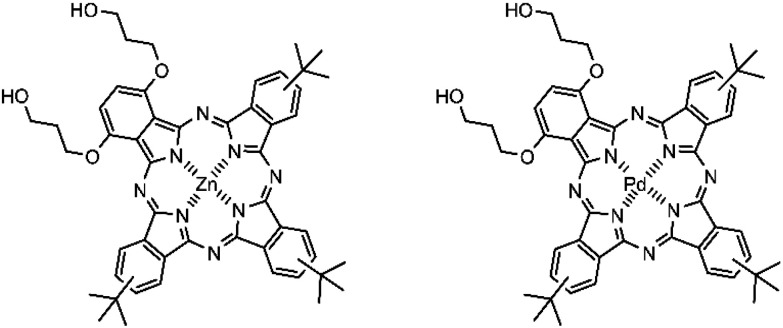
Chemical structures of ZnBu_3_ProH_2_ and PdBu_3_ProH_2_.

Several other metals and metal complexes have been investigated for biomedical applications such as chemotherapeutic, antimicrobial, and antifungal agents. Among these, zinc(ii) and its complexes represent a promising alternative to palladium due to their abundance and lower cost. Zinc is the second most prevalent transition metal in the human body.^32,35^ Zinc phthalocyanines (ZnPc) and their derivatives have been extensively explored for PDT, with some reaching clinical use and commercialization.^36,37^ In this work, we compared the release profiles of ROS-sensitive liposomes encapsulating two structurally identical phthalocyanines differing only in their central metal PdBu_3_ProH_2_ and ZnBu_3_ProH_2_ based on the abovementioned criteria (Fig. 3).

The lipid formulation consisted of DOTAP : DSPE-PEG : cholesterol in a molar ratio of 45 : 50 : 5. Our previous work demonstrated that PdBu_3_ProH_2_-loaded liposomes could effectively release both macromolecules (rhodamine B-dextran) and small molecules (calcein) upon red-light irradiation at 630 nm.^31^ However, considering the limitations of palladium for drug-delivery applications, namely, cost and potential toxicity, we substituted palladium with zinc and evaluated the release from ZnBu_3_ProH_2_-loaded ROS-sensitive liposomes under 690 nm irradiation.

**Fig. 4:**
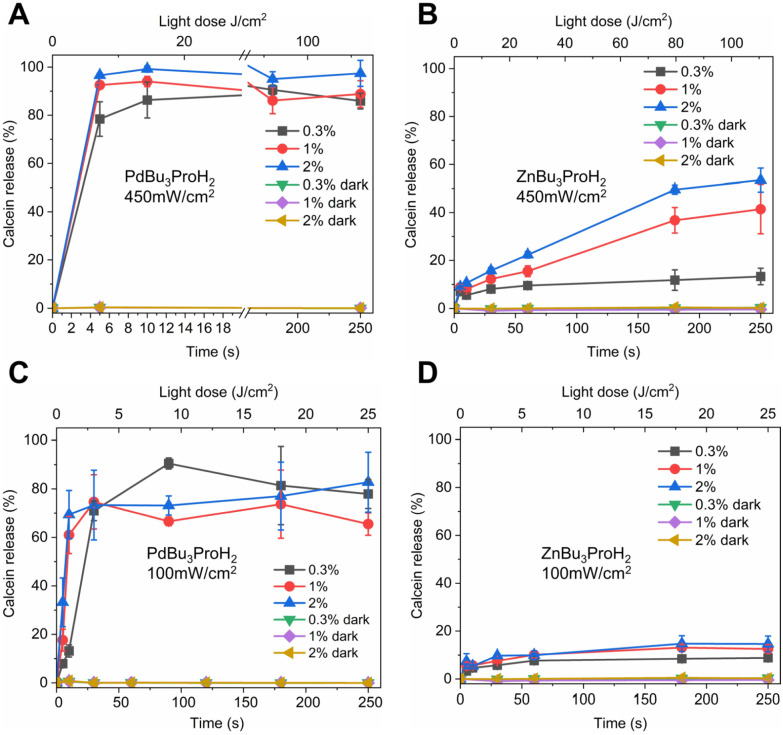
Calcein release from ZnBu_3_ProH_2_- and PdBu_3_ProH_2_-loaded liposomes after illumination with a 690 nm laser at 450 and 100 mW cm^−2^ under aerobic conditions. (A) Calcein release from PdBu_3_ProH_2_-loaded liposomes during illumination with laser at 450 mW cm^−2^. (B) Calcein release from ZnBu_3_ProH_2_-loaded liposomes during illumination with laser at 450 mW cm^−2^. (C) Calcein release from PdBu_3_ProH_2_-loaded liposomes during illumination with laser at 100 mW cm^−2^. (D) Calcein release from ZnBu_3_ProH_2_-loaded liposomes during illumination with laser at 100 mW cm^−2^.

**Fig. 5:**
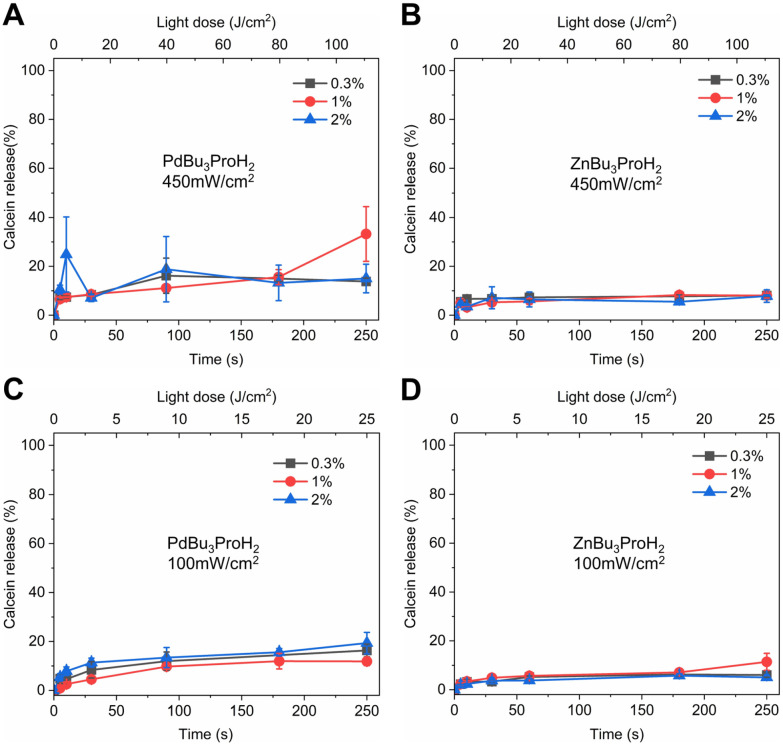
Calcein release from ZnBu_3_ProH_2_- and PdBu_3_ProH_2_-loaded liposomes after illumination with a 690 nm laser at 450 and 100 mW cm^−2^ under anaerobic conditions. (A) Calcein release from PdBu_3_ProH_2_-loaded liposomes during illumination with laser at 450 mW cm^−2^. (B) Calcein release from ZnBu_3_ProH_2_-loaded liposomes during illumination with laser at 450 mW cm^−2^. (C) Calcein release from PdBu_3_ProH_2_-loaded liposomes during illumination with laser at 100 mW cm^−2^. (D) Calcein release from ZnBu_3_ProH_2_-loaded liposomes during illumination with laser at 100 mW cm^−2^.

Both photosensitizers exhibit strong absorption in the far-red region, with maximum extinction coefficients of 185 000 M^−1^ cm^−1^ at 690 nm for PdBu_3_ProH_2_ and 187 900 M^−1^ cm^−1^ at 695 nm for ZnBu_3_ProH_2_ in toluene. The Soret band maxima of these metal phthalocyanines are located between 330 and 350 nm (Fig. S1, SI). Illumination experiments were carried out using our custom-built irradiation system under both aerobic and anaerobic conditions in 20 mM, pH 7.4 HEPES buffer at 37 °C (Fig. 4 and 5). All experiments were performed in white 96-well plates to ensure constant temperature conditions. Preliminary measurements indicated that black 96-well plates absorb incident light and undergo heating up to ∼20 °C, which could interfere with experimental accuracy (Fig S2, SI). Therefore, white plates were used in this study and are also recommended for any similar studies in the future. Illumination of 0.3 mol%–2 mol% PdBu_3_ProH_2_ loaded liposomes with 690 nm and 450 mW cm^−2^ power density lasers for 10 seconds (4.5 J cm^−2^) was sufficient to release at least 80% of calcein under normal oxygen conditions. In contrast, calcein release from 0.3 mol%–2 mol% ZnBu_3_ProH_2_ loaded liposomes after 10 seconds of illumination reached only 10%. After illumination for 250 seconds (112.5 J cm^−2^), the maximum value of the calcein released from liposomes loaded with zinc phthalocyanine was 50%. Under anaerobic conditions, illumination with a 450 mW cm^−2^ power density laser showed that the release of calcein from both PdBu_3_ProH_2_ and ZnBu_3_ProH_2_ loaded liposomes was significantly lower than under aerobic conditions, between 10 and 20% (Fig. 5A and B). This demonstrates the oxygen dependent nature of the release mechanism, indicating the generation of oxygen species by both photosensitizers as the main driver for release from liposomes. After decreasing the laser's power density to 100 mW cm^−2^, the release of calcein for both liposomes also slowed down. The maximum release for PdBu_3_ProH_2_ after irradiating for 250 seconds (25 J cm^−2^) was around 80% and for ZnBu_3_ProH_2_ it was 15%. In both cases, light-induced calcein release increased with the loading percentage of the photosensitizer in the liposomal formulation. The higher loading of the photosensitizer leads to the generation of a higher concentration of reactive oxygen species.^31^ The experiment was repeated under anaerobic conditions, where calcein release remained below 20–30% for both PdBu_3_ProH_2_ and ZnBu_3_ProH_2_ loaded liposomes (Fig. 5C and D), again confirming the ROS sensitivity of the liposomes.

The size of liposomes loaded with photosensitizers was measured before and after illumination with a 450 mW cm^−2^ power density laser (Table 1). The results showed that there is no significant difference in the liposomal size. This suggests that illumination-induced oxidation of DOTAP alters membrane hydrophobicity or promotes pore formation while maintaining overall liposomal integrity.^31^ However, ZnBu_3_ProH_2_ liposomes consistently showed much lower release compared to PdBu_3_ProH_2_ liposomes. This difference can be attributed to the lower encapsulation efficiency of zinc phthalocyanine, which varied between 3 and 9% depending on the initial feeding amount, whereas the encapsulation efficiency for palladium phthalocyanine was approximately four to five times higher, 20–35% (Table S2, SI). As the two molecules are very similar, this difference in encapsulation efficiencies is surprising. One reason could be in the fine details of the location of the central metal ion and its crystal field configuration. Palladium(ii) is a d^8^ system and typically forms square-planar complexes, whereas zinc(ii), with a d^10^ configuration, more commonly adopts a tetrahedral or octahedral geometry.^38,39^

**Table 1:** Size of liposomes loaded with PdBu_3_PrOH_2_ and ZnBu_3_ProH_2_ before and after illumination with a 690 nm laser at a power density of 450 mW cm^−2^

Time (s)	0.3 mol%		1 mol%		2 mol%	
	Size (nm)	PDI	Size (nm)	PDI	Size (nm)	PDI
PdBu_3_ProH_2_						
0	133.6	0.12	124.8	0.09	125.4	0.09
5	129.7	0.07	120	0.09	134.3	0.07
300	123.9	0.07	116.6	0.09	118.5	0.10
ZnBu_3_ProH_2_						
0	125.3	0.10	129	0.08	118.2	0.06
10	139.8	0.19	135	0.19	134.3	0.07
300	131.5	0.14	134.1	0.19	127.4	0.23

Theoretical studies on the planarity of unsubstituted zinc phthalocyanine (ZnPc) have shown that a planar structure is preferred in the gas phase. However, it was shown that ZnPc favors binding of additional axial ligands; in the monohydrated complex (ZnPc–H_2_O), the zinc ion is displaced by ∼0.4 Å out of the ligand plane.^40^ In contrast, palladium complexes with a square-planar geometry do not readily bind axial ligands. Based on this, we assume that during the hydration step in liposome preparation, ZnBu_3_ProH_2_ may interact with water molecules, which could reduce its encapsulation efficiency within the lipid bilayer. The singlet oxygen quantum yields of ZnBu_3_ProH_2_ and PdBu_3_ProH_2_ were 50% and 67%, respectively, indicating that the lower ROS generation of ZnBu_3_ProH_2_ may also contribute to its reduced calcein release from liposomes. Additionally, the absorption spectra of liposomes encapsulating varying concentrations of phthalocyanines were measured to evaluate potential aggregation with increasing concentration. The results showed no detectable blue shift in the absorption maxima, indicating minimal to no aggregation of phthalocyanines within the liposomal bilayer as their concentration increased (Fig. S5). Overall, PdBu_3_ProH_2_ exhibited a superior calcein release profile compared to ZnBu_3_ProH. The cheaper ZnBu_3_ProH_2_ could still be useful but would require the use of a relatively higher amount of the dye during the encapsulation process. Although we were able to compare the effect of the metal on the cargo release profile from ROS-sensitive liposomes using our custom illumination system, further analysis is required to fully understand the different impacts of the metal on cargo release. The data itself were reproducible, and our illumination system itself functioned reliably throughout the studies, as indicated by the relatively small error values, which are most likely largely impacted by variations in the liposome preparation as well.

## Materials and methods

### Materials

1,2-Dioleoyl-3-trimethylammonium-propane (DOTAP) and 1,2-distearoyl-*sn-glycero*-3-phosphoethanolamine-N [maleimide(polyethylene glycol)-2000] (DSPE-PEG) were purchased from Avanti Polar Lipids (USA). Cholesterol, HEPES buffer, chloroform, Triton X-100 (10% solution), calcein, sodium sulfite (Na_2_SO_3_), sodium hydroxide (NaOH), formic acid, ammonium formate, sodium tetrachloropalladate(ii), pyridine, methanol (MeOH), acetonitrile, silica 60, silica 100, dichloromethane (DCM), ethanol, 3,6-di(hydroxypropyloxy)phthalonitrile, and 4-*tert*-butylphthalonitrile were purchased from Merck (Germany). Rhodamine B dextran was ordered from Thermo Fisher Scientific (USA). All chemicals and solvents were used as received.

#### Custom illumination setup and control software

A custom illumination setup was developed by extensively modifying a commercial laser engraving system (VEVOR L3030, China). The original device features a working area of 410 × 400 mm and is constructed from an anodized aluminum alloy for enhanced durability and structural stability. Its compatibility with GRBL v1.1 open-source firmware—operable across Windows (7/8/10/11), iOS, and Android platforms—enabled seamless integration with a custom-developed control application written in Python. To repurpose the system for precise optical illumination experiments, substantial hardware modifications were performed. The default laser module was removed from the moving head and replaced with a 3D-printed custom lens tube holder (4 × 4 cm, central aperture diameter 2.5 cm designed by SolidWorks, SI). A fiber-coupled 690 nm laser source (Roithner Laser Technik, Austria) with a maximum output power of 1200 mW was integrated using a multimode optical fiber patch cable (Thorlabs M152l02; 400 μm core diameter, 0.22 NA, SMA-SMA connectors). The fiber terminates inside the lens tube (Thorlabs SM1L10) with the fiber adaptor (Thorlabs SM1SMA), where an aspheric condenser lens (Thorlabs ACL1815U; 15 mm focal length) collimates the laser beam for consistent and precise illumination. To further control the spatial profile of the beam, a variable mechanical iris diaphragm (Thorlabs D36S; adjustable aperture ∅ 1.9 mm–36.0 mm) was incorporated into the lens tube assembly, enabling dynamic modulation of beam diameter prior to reaching the sample. The collimated beam is precisely guided to individual wells of a standard 96-well plate (or 24-well plate) placed on an Eppendorf ThermoMixer C equipped with a temperature-controllable SmartBlock plate, ensuring stable and uniform thermal conditions throughout illumination experiments.

The lens holder is mounted on a manually adjustable *Z*-axis stage, allowing vertical tuning up to 12 cm to enable accurate beam focusing. The entire assembly is placed on a 22 cm height-adjustable platform, stabilized with threaded leveling rods for mechanical robustness and positional flexibility. The system retains its original *X*–*Y* motion control capabilities, powered by three integrated stepper motors and governed by the VEVOR controller box, now operated with a positional accuracy of 0.1 mm in both axes. Laser power modulation was achieved using a high-precision, large-core fiber variable attenuator (Agiltron HPLA-111010238), compatible across the full wavelength range and controlled *via* our Python-based software. To ensure optical isolation and eliminate ambient light interference, the complete setup is enclosed in a light-tight black box made of plexiglass. The total cost of constructing this custom illumination platform—excluding the laser source and any thermal shaking/incubation components—was approximately 458 euros, making it an affordable and accessible alternative to commercial illumination systems (total cost provided in the SI).

To complement the hardware, a dedicated Python application—AIS Illumination software—was developed to manage all device functions. Built using the PyQt6 framework, the graphical interface enables intuitive operation and is supported by multithreading and multiprocessing to maintain real-time responsiveness during motor control and laser actuation. Communication with the device is established *via* a serial protocol, ensuring robust and low-latency command execution. A comprehensive technical description of the software, detailed cost analysis, and links to the code and CAD models are provided in the SI.

### Liposome preparation

Liposomes were prepared based on the procedure described previously. Briefly, DOTAP and DSPE-PEG were dissolved in chloroform, and mixed in a molar ratio of 45 : 5 : 50. Subsequently, 0.3–2 mol% of PdBu_3_ProH_2_ or ZnBu_3_ProH_2_ dissolved in chloroform was added. The mixture was evaporated in a rotavapor for 30 min at 67 °C at 10 mbar. The thin lipid film was hydrated with 1 ml of calcein solution (60 mM, 280 mOsm, pH 7.4) or with 1 ml of HEPES buffer (20 mM HEPES, 140 mM NaCl, pH 7.4). Liposomes without calcein were used for further quantification of encapsulated phthalocyanines. The suspension was hydrated at 67 °C until full solubility of the thin film. The liposomes were extruded 13 times with a 100 nm pore size polycarbonate membrane. Finally, liposomes were purified by using size-exclusion chromatography (SEC) on a Sephadex G-50 gel filtration medium and eluted with HEPES buffer.

### Characterization of the calcein loaded liposomes

The size of the liposomes was measured at different illumination times (0, 5, 10, and 300 s) with dynamic light scattering (DLS) using a Zetasizer Nano Series instrument, Malvern Instruments (United Kingdom).

### Calcein release from liposomes

The purified liposomes were diluted with HEPES buffer (1 : 10, pH 7.4). The liposomes were illuminated with 690 nm, 100 mW cm and 450 mW cm^−1^ at 37 °C using a custom high-throughput illumination setup for 5–300 seconds. Dark control samples in the same 96-well plate were shielded from the light. Triton-X (10%, 10 µl) was used to determine the maximum release of calcein from liposomes. The fluorescence of the calcein was measured using a Varioskan Lux plate reader, Thermo Fisher Scientific (USA), at excitation and emission wavelengths of 493 and 518 nm, respectively. Each experiment was conducted in triplicate and the average value and standard deviation were calculated. The percentage of released calcein was calculated by using eqn (1).1
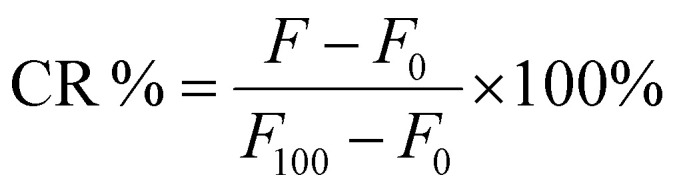
where *F* is the fluorescence of the sample at a specific measurement point, *F*_0_ is the background fluorescence of the cold control sample, and the *F*_100_ is the maximum release of calcein following the addition of 10% Triton-X. CR% is the calcein release percentage.

### Calcein release from liposomes under anaerobic conditions

To study the calcein release in a hypoxic environment, a solution of sodium sulfite (18 mM) was used. Sodium sulfite (Na_2_SO_3_) is a molecular oxygen scavenger that inhibits reactive oxygen species generation by depleting ground-state oxygen.

### Synthesis of 1,4-di[hydroxypropyloxy]-9(10),16(17),23(24) tri[*tert*-butyl]phthalocyaninato (2-)-N29,N30,N31,N32 zinc(ii), ZnBu_3_PrOH_2_

Zinc acetate dihydrate (50 mg, 0.136 mmol) and 1,4-di[hydroxypropyloxy]-9(10),16(17),23(24)tri[*tert*-butyl]phthalocyanine (300 mg, 0.06 mmol) were dissolved in fresh dimethylformamide (10 ml) and the reaction was heated at reflux overnight under an argon atmosphere. The solution was cooled to room temperature, evaporated on a rotatory evaporator, and washed with chloroform. After washing, the collected product was purified by column chromatography on Silica 60 and eluted with neat chloroform stabilized with EtOH. After short column chromatography, the collected fraction was purified by preparative TLC, eluting with CHCl_3_ : EtOH 18 : 1. The collected product was obtained as a dark green powder. UV-vis: *λ*_max_(toluene)/346 nm (*ε*/dm^3^ mol^−1^ cm^−1^ 92 500), 695 (195 000). NMR: *δ*_H_ (500 MHz; CDCl_3_/CD_3_OD 10 : 1; Me4Si) 9.5–9.1 (6 H, m, phthalo-H), 8.25–8.1 (3 H, m, phthalo-H), 7.57–7.4 (2 H, m, 2,3-phthalo-H), 4.9–4.6 (8 H, m, OCH_2_CH_2_CH_2_OH), 2.2–2.0 (4 H, m, OCH_2_CH_2_CH_2_OH), 1.78–1.69 (27 H, m, C(CH_3_)_3_). MS: *m*/*z* 893.348756 ((M + H)^+^) (calcd for C_50_H_52_N_8_O_4_Zn 893.3475).

### 1,4-Di[hydroxypropyloxy]-9(10),16(17),23(24) tri[*tert*-butyl]phthalocyaninato (2-)-N29,N30,N31,N32 palladium(ii), PdBu_3_PrOH_2_

PdBu_3_PrOH_2_ was synthesized using the same method as previously described.^31^

### Singlet oxygen quantum yield measurement

Singlet oxygen luminescence was measured using a time-correlated single-photon counting (TCSPC) system, Fluotime 300 (Pico-Quant GmBH, Chaussee, Germany) consisting of a PicoHarp 330 controller and a PDL 820 driver. The samples were placed in a 1 cm^2^ SOG cuvette and excited with the pulsed diode laser head LDH-D-C-640 at 640 nm. The emission of the generated singlet oxygen was recorded using a near-infrared photomultiplier tube (NIR PMT, Hamamatsu, Japan) in the 1220–1350 nm range, with a distinct maximum at 1270 nm.

Conventional unsubstituted Zn phthalocyanine (ZnPc) with known singlet oxygen quantum yield in toluene of 58%^41^ was used as a reference to determine the quantum yield of singlet oxygen of PdBu_3_ProH_2_ and ZnBu_3_ProH_2_. For that, the absorption spectra of four samples each of PdBu_3_ProH_2_, ZnBu_3_ProH_2_, and ZnPc in toluene were recorded. The samples were excited at 640 nm, and the emission of singlet oxygen was measured. The integrated emission intensity (from 1220 to 1350 nm) was plotted against the absorption of a sensitizer at 640 nm. The singlet oxygen quantum yield was then calculated using the following formula:2
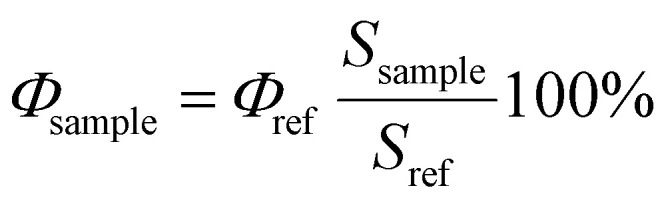
where, *Φ*_sample_ is the singlet oxygen quantum yield of the sample in toluene, *Φ*_ref_ is the singlet oxygen quantum yield of the ZnPc in toluene (58%), *S* is the slope of the plot of absorbance at 640 nm *vs.* the integrated emission intensity.

## Conclusions

The development of light-responsive drug delivery systems requires an experimental setup that ensures reproducibility precision, scalability, and cost-effectiveness. In this study, we addressed this demand by designing a custom-built illumination platform that is compatible with standard well plate formats, incorporates a temperature-controlled thermoshaker for uniform conditions, and enables automated well selection with 0.1 mm accuracy *via* Python-based AIS Illumination software. The optical configuration, consisting of a fiber-coupled laser, adjustable iris, and collimating optics, provides precise control over beam diameter and irradiance, making the system adaptable for 96-, 24-, 12-, and 384-well plates. With a tunable output ranging from 10 mW to 1200 mW, the setup allows experiments across a broad range of light intensities. Together, these features establish a versatile and affordable tool for high-throughput *in vitro* studies of light-activated drug delivery.

Using this platform, we directly compared the performance of ROS-sensitive liposomes loaded with structurally identical phthalocyanines differing only in their central metal ion, PdBu_3_ProH_2_ and ZnBu_3_ProH_2_. Despite both photosensitizers absorbing in the red/far-red region (690 nm), PdBu_3_ProH_2_ showed better performance than ZnBu_3_ProH_2_. It exhibited a higher singlet oxygen quantum yield (67% *vs.* 50%), greater encapsulation efficiency (20–35% *vs.* 3–9%), and significantly faster and more complete calcein release (up to 100% within 10–250 s, compared to ≤50% for ZnBu_3_ProH_2_ under the same conditions). The significantly reduced release under anaerobic conditions confirmed the ROS-dependent nature of the mechanism. Importantly, dynamic light scattering revealed no significant changes in liposome size before and after irradiation, indicating that light-triggered oxidation alters membrane permeability without compromising vesicle integrity. The inferior performance of ZnBu_3_ProH_2_ can be rationalized by both its lower loading efficiency and its structural tendency to bind axial ligands in aqueous environments, which reduces compatibility with the lipid bilayer. In contrast, the square-planar geometry of PdBu_3_ProH_2_ resists axial coordination and promotes more stable encapsulation. These differences illustrate how the choice of central metal strongly influences the physicochemical and photochemical properties of phthalocyanine photosensitizers in drug delivery applications.

In summary, our results demonstrate that PdBu_3_ProH_2_-loaded ROS-sensitive liposomes exhibit superior performance over their ZnBu_3_ProH_2_ counterparts; however, palladium costs higher. Thus, using our custom illumination system, we were able to reproduce and compare calcein release from different liposomes within short periods of time. Our results demonstrate its practical utility for diverse photochemical and photobiological studies.

## Author contributions

Ali Eftekhari: conceptualization, methodology, investigation, visualization, software, and writing – original draft; Olga Lem: methodology, formal analysis, investigation, writing – original draft, and visualization; Timo Laaksonen, Nikita Durandin, Alexander Efimov: conceptualization, supervision, funding acquisition, and writing – review & editing.

## Conflicts of interest

There are no conflicts to declare.

## Supplementary Material

AN-150-D5AN00927H-s001

## Data Availability

The data supporting the manuscript have been included as part of the supplementary information (SI). Supplementary information is available. See DOI: https://doi.org/10.1039/d5an00927h. The code for the automated control of the setup described in the manuscript is available in the folder of the GitHub repository at https://github.com/Eftekhari92/Ali-illumination.
