# Supplementary material for: An automated illumination system for high-throughput photopharmacology studies: a case study of ROS-sensitive Zn- and Pd-phthalocyanine-loaded liposomes
Source: Analyst. 2025 Oct 20;150(24):5473–80. doi: 10.1039/d5an00927h (PMC12612984; doi:10.1039/d5an00927h)
Supplement: AN-150-D5AN00927H-s001 [file AN-150-D5AN00927H-s001.pdf]

Supplementary Information (SI) for Analyst.
This journal is © The Royal Society of Chemistry 2025

Supporting Information

An automated illumination system for high-throughput photopharmacology studies: ROS-sensitive Zn- and Pd-phthalocyanine-loaded liposomes case study

Ali Eftekhari[a#], Olga Lem[a#], Alexander Efimov[a] , Timo Laaksonen[a,b*], Nikita Durandin[a*]*

Loading efficiency and encapsulation efficiency of photosensitizers:

The loading efficiency based on the average of the experiments was defined as Equation 1. states (Equation 2.). The actual amount of photosensitizer encapsulated in the liposomes was determined by measuring the absorption. The liposomes not loaded with calcein were diluted with pyridine in the 1:3 ration respectively. Based on the absorption values of the photosensitizer, the mass was determined.

$$LE\% = \frac{M_{actual}}{M_{total}} x100\%$$

Where LE% is the loading efficiency, M_{actual} is the actual amount of photosensitizer encapsulated in the liposomes, M_{total} is the total mass of the photosensitizer and lipids in liposomes.

By using the determined concentration of the photosensitizer, the encapsulation efficiency was calculated using the following equation (3):

$$EE\% = \frac{C_{actual}}{C_{theoretical}} x\ 100\%$$

Where EE% is the encapsulation efficiency, C_{actual} is the maximum concentration measured to be encapsulated in the liposomes, $C_{theoretical}$ is the original concentration of the loaded phthalocyanines.

Comparison of white and black 96 well-plates

The well plates were filled with the 200 ul of HEPES buffer. Each well was illuminated with 690 nm, 450 mWcm^{-1} at 37 ˚C using custom high-throughput illumination setup for 5-400 seconds. To analyze the temperature difference, measurements were taken before and right after the illumination by the wired thermometer.

Custom Illumination Control Software

To complement the hardware, a dedicated Python application—AIS Illumination software—was developed to manage all device functions. Built using the PyQt6 framework, the graphical interface

enables intuitive operation and is supported by multithreading and multiprocessing to maintain real-time responsiveness during motor control and laser actuation. Communication with the device is established via serial protocol, ensuring robust and low-latency command execution.

The AIS Illumination software comprises four main functional panels:

1. **Connection Panel**: Allows users to select the appropriate COM port and baud rate to establish communication with the device. A red status indicator and “Connected” message confirm a successful connection.
2. **Initialization Panel**: Used to define the system’s reference coordinates. The Home and A1 well positions can be established either by direct coordinate input or through manual adjustment using directional arrow buttons. Step size is configurable between 0.1 mm and 400 mm. This calibration is performed once during initial setup and stored for future use.
3. **Coordination Panel**: Facilitates selection of target wells (A1–H12) and the definition of illumination durations ranging from 1 second to several hours per well. This panel also includes controls for a shutter and iris, allowing the beam size to be adjusted or blocked entirely. Errors or operational messages are reported in a dedicated command console.
4. **G-Code Panel**: Displays the full sequence of G-code commands generated by the system, providing users with detailed insight into the control logic and facilitating reproducibility or manual adjustments if necessary.

Data availability

To promote reproducibility and facilitate future adaptations by the research community, the complete source code for the Ali Illumination control software (AIS) is freely available on GitHub at https://github.com/Eftekhari92/Ali-illumination. Additionally, all custom-designed 3D-printable CAD files used in the hardware modification are provided at https://github.com/Eftekhari92/Cad-files.

Table S1. Total final price in Finland (08.08.2025).

Item	Price (in euro)
Illumination setup	458
A fiber-coupled 690 nm laser source (Roithner Laser Technik, Austria)	5720
Thermomixers with 96-well plate Eppendorf	3200
Agiltron laser attenuator	1250
Total	10628

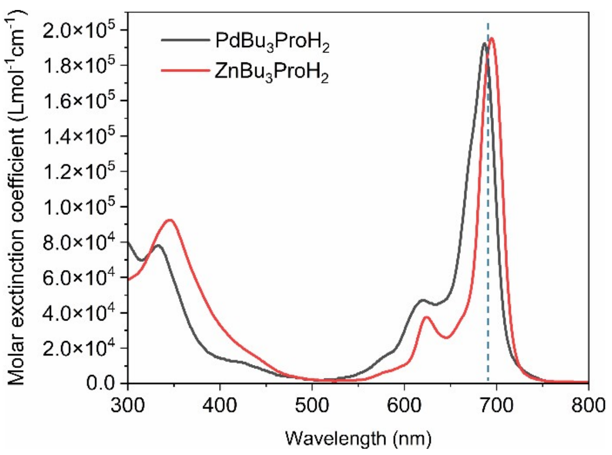

Fig.S1 Absorption of $PdBu_3ProH_2$ and $ZnBu_3ProH_2$ in toluene.

Table S2. Encapsulation efficiency and loading efficiency of $PdBu_3ProH_2$ and $ZnBu_3ProH_2$ loaded in liposomes

Encapsulation efficiency,%			Loading efficiency,%		
$PdBu_3ProH_2$					
0.3 mol%	1 mol%	2 mol%	0.3 mol%	1 mol%	2 mol%
35.5±1.0	25.7 ±0.5	20.6±0.7	0.2±0.01	0.4±0.01	0.6±0.02
$ZnBu_3ProH_2$					
9.3±0.5	6.6±0.4	3.4±0.1	0.02±0.001	0.05±0.004	0.05±0.002

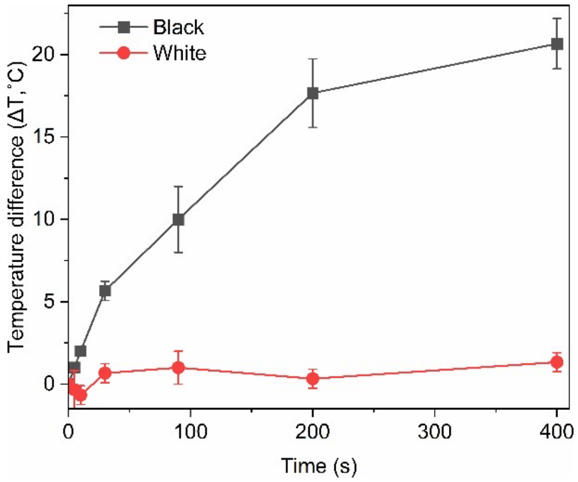

Fig. S2 Temperature profiles of black and white 96-well plates under 690 nm laser illumination (450 mW cm^{-2}, 0–400 s)

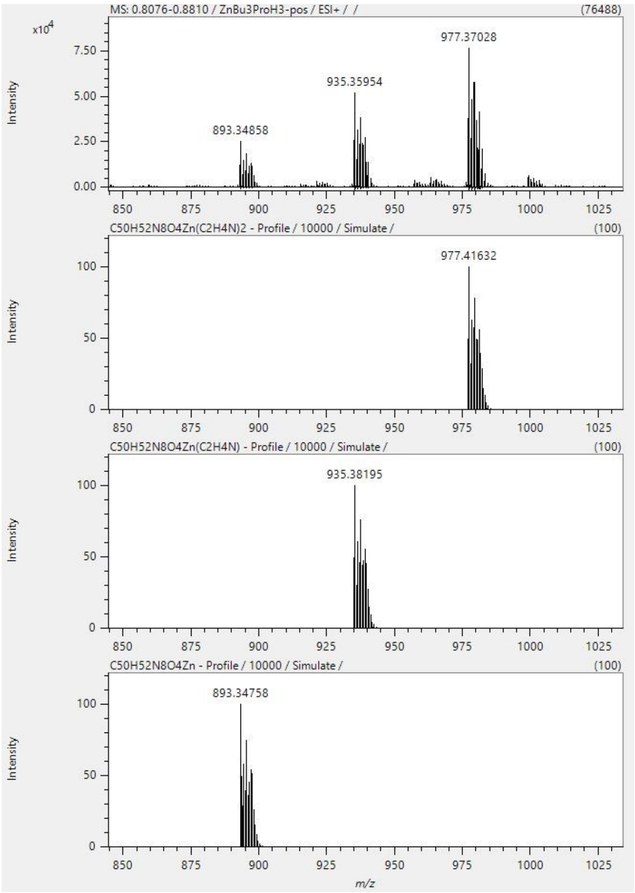

Fig.S3 Mass spectra of $ZnBu_3ProH_2$

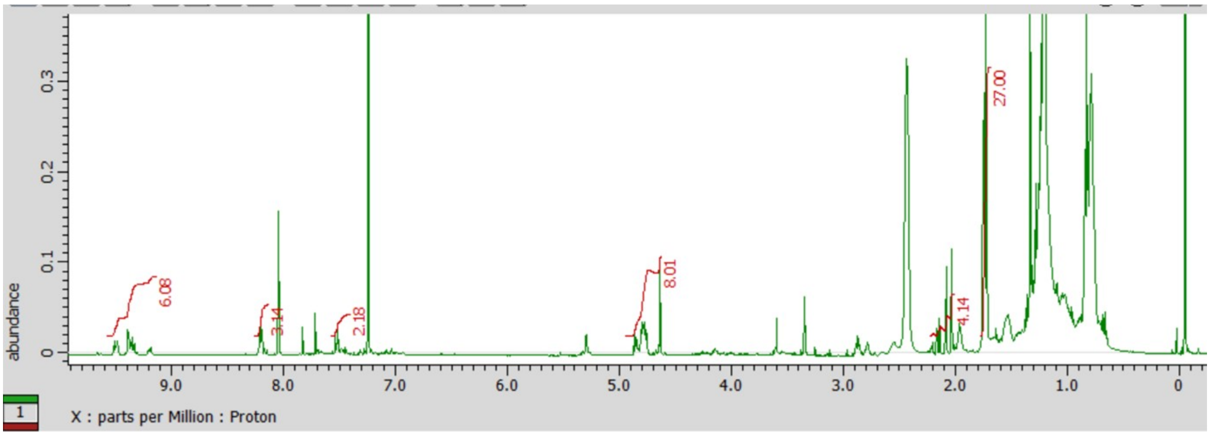

Fig.S4 NMR of $ZnBu_3ProH_2$

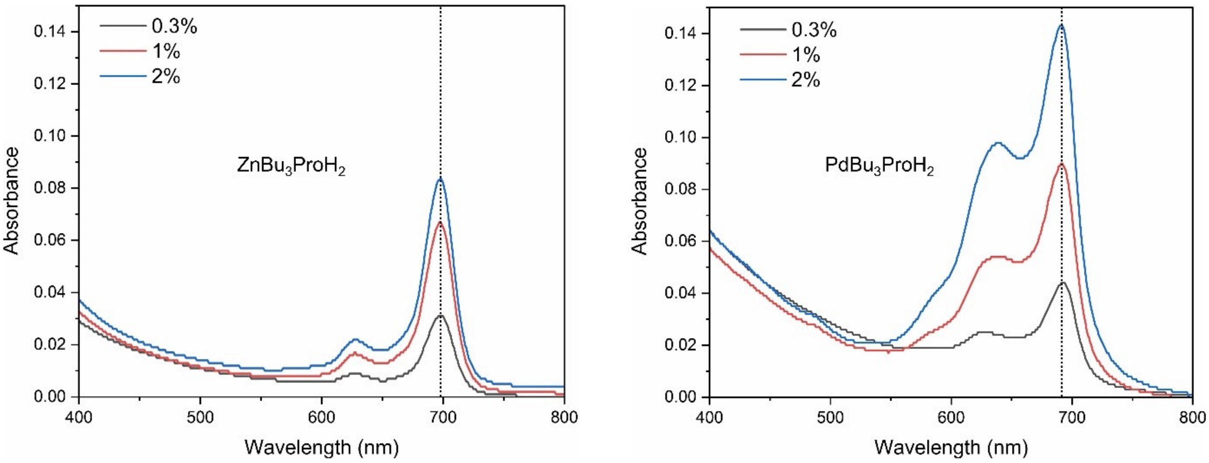

Fig.S5 Absorption of ZnBu$_3$ProH$_2$ and PdBu$_3$ProH$_2$ encapsulated in liposomes

Table S3. Peak ratio of at 627: 698 nm for ZnBu$_3$ProH$_2$ and at 635:690 nm for PdBu$_3$ProH$_2$ encapsulated in liposomes

Loading	ZnBu$_3$ProH$_2$ peak ratio at 627:698 nm	PdBu$_3$ProH$_2$ peak ratio at 635:690 nm
0.3%	3.4	1.76
1%	3.9	1.6
2%	3.8	1.46
